# Antibiotic Resistance Genes in Wastewater: A Systematic PRISMA-Guided Review on Risk, Genetic Transfer, and the Effectiveness of the Photo-Fenton Process for Their Removal

**DOI:** 10.3390/jox16030094

**Published:** 2026-05-25

**Authors:** María del Rocío Duarte-Martínez, Aldo Amaro-Reyes, Juan Campos-Guillen, Miguel Angel Ramos-López, Eloy Rodríguez-de León, Monserrat Escamilla-García, Vanessa Vallejo-Becerra, Alejandra Álvarez-López, Yesenia Mendoza-Burguete, Mónica López Velarde-Santos, Héctor Pool, Luisa Ramírez-Granados, Ricardo Chaparro-Sánchez, José Alberto Rodríguez-Morales

**Affiliations:** 1 Centro de Investigación en Materiales Semiconductores, Sustentabilidad y Energía Renovable (CIMSSER), Facultad de Química, Universidad Autónoma de Querétaro, Cerro de las Campanas S/N, Las Campanas, Queretaro 76010, Mexico; mduarte19@alumnos.uaq.mx (M.d.R.D.-M.);; 2 Facultad de Química, Universidad Autónoma de Querétaro, Cerro de las Campanas S/N, Las Campanas, Queretaro 76010, Mexico; 3 División de Estudios de Posgrado, Facultad de Ingeniería, Universidad Autónoma de Querétaro, Cerro de las Campanas S/N, Las Campanas, Queretaro 76010, Mexicomaria.yesenia.mendoza@uaq.mx (Y.M.-B.); monica.lopez.velarde.santos@uaq.mx (M.L.V.-S.);; 4 Facultad de Informatica, Universidad Autónoma de Querétaro, Av. de las Ciencias s/n, Juriquilla, Campus Juriquilla, Queretaro 76010, Mexico

**Keywords:** advanced oxidation processes, antimicrobial resistance, wastewater, horizontal gene transfer

## Abstract

Antimicrobial resistance (AMR) constitutes a growing global threat, facilitated by the dissemination of antibiotic resistance genes (ARGs) through wastewater treatment plants (WWTPs). This systematic review, conducted following the PRISMA guidelines, compiles the risks associated with ARGs, as well as the factors that promote horizontal gene transfer (HGT) and the technologies applied for their removal. The literature shows that WWTPs act as reservoirs, where biological treatment conditions and the presence of sub-inhibitory contaminants (antibiotics, metals, and pharmaceuticals) accelerate HGT. Although conventional methods (chlorination, ozonation, UV) are effective at eliminating antibiotic-resistant bacteria (ARB), their ability to degrade persistent genetic material is insufficient. Therefore, advanced oxidation processes (AOPs) emerge as a key solution, with the photo-Fenton process standing out due to efficiently generating hydroxyl radicals, achieving the degradation of ARGs, an essential step to mitigate the spread of AMR into the environment.

## 1. Introduction

Antibiotics are drugs designed to eliminate or inhibit the growth of microorganisms and are therefore widely used to treat a variety of infections. However, the excessive and inappropriate use of these compounds has promoted the development of antibiotic-resistant bacteria (ARB) [1]. This resistance represents an increasing global public health concern, as bacteria develop the ability to withstand antibiotics that were previously effective [2,3]. The consequences of this situation include increased mortality, prolonged recovery times, and higher healthcare costs [4,5]. The World Health Organization has further estimated that, if left uncontrolled, bacterial resistance could result in approximately 10 million deaths worldwide. In addition, it has been reported that subinhibitory concentrations of antibiotics present in the environment may significantly contribute to the development of this resistance [1]. In this context, human consumption represents the main route through which antibiotics enter the environment, due to both the excretion of unmetabolized drugs and the improper disposal of expired medications, which are often discharged directly into sewer systems [6]. Moreover, the global rise in antibiotic consumption and their widespread availability have resulted in their occurrence in diverse environmental sources, including veterinary, agricultural, aquacultural, and pharmaceutical applications [7,8].

Consequently, the inappropriate use of antibiotics has significantly contributed to the dissemination of antibiotic resistance genes (ARGs). These genes can be transmitted through both vertical and horizontal gene transfer, enabling their widespread distribution across different ecosystems [9]. In this regard, numerous studies have demonstrated that ARGs can be transmitted between bacterial species through mobile genetic elements, including plasmids, integrons, and transposons, thereby enabling their rapid spread [10,11]. Consequently, ARGs are regarded as emerging contaminants of significant concern, as they undermine the efficacy of clinical treatments for bacterial infections [12].

Wastewater treatment plants (WWTPs) are designed to reduce organic matter to acceptable levels; however, they do not completely eliminate resistant bacteria or their genes [13]. In fact, they act as reservoirs of ARGs and provide a favorable environment for their dissemination due to high microbial density and the simultaneous presence of diverse contaminants [14]. In particular, biological treatment creates conditions favorable for horizontal gene transfer (HGT) by bringing together a wide variety of bacteria in a nutrient-rich environment exposed to antibiotics or their residues [3]. Even low concentrations of these compounds (ng/L–μg/L) in bioreactors can select for resistant bacteria and facilitate the spread of ARGs to initially sensitive bacteria [15]. Since treated effluents are discharged into surface waters, WWTPs play a crucial role in the dissemination of resistant bacteria and genes into the environment, and consequently, to humans [15].

Traditional secondary treatments, such as activated sludge, are largely ineffective at biological risk mitigation, as they often promote the concentration of ARGs through horizontal gene transfer in the biomass. In direct contrast, advanced oxidation processes (AOPs) do not rely on biomass and therefore avoid this unintended enrichment of resistance genes. Even traditional disinfection methods like chlorination show significant limitations; they may induce cellular stress and select for tolerant bacterial strains without degrading the underlying extracellular genetic material. AOPs, however, generate non-selective radicals, primarily hydroxyl radicals (•OH), capable of mineralizing the DNA backbone and providing a permanent solution to the dissemination of genetic material.

**Figure 1:**
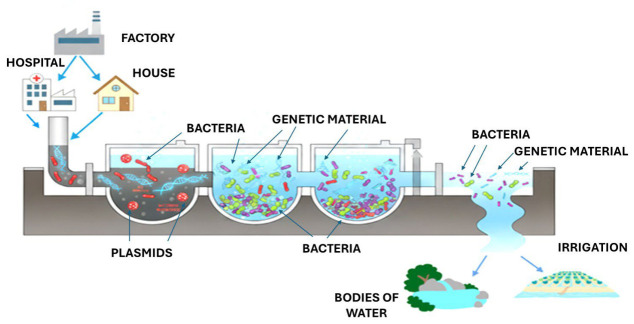
Pathways of antibiotic-resistant bacteria and antibiotic resistance genes entry into water bodies and soil.

Figure 1 illustrates the process by which antibiotics enter wastewater through various sources, including domestic, hospital, and industrial discharges. The continuous presence of these compounds in the sewer system promotes the selection and proliferation of ARGs. These genes persist within WWTPs, where they can be maintained or even amplified due to process conditions. Finally, the treated effluent, which may still contain resistance genes, is discharged into the environment, contributing to their dissemination.

Although the removal of organic substrates and nutrients is the primary focus of WWTPs, the elimination of emerging contaminants such as ARGs remains insufficient and requires greater attention [16].

While disinfection processes used in wastewater treatment remove most pathogens and have been shown to be effective against resistant bacteria, intact DNA can persist even after bacterial inactivation [16]. These limitations demonstrate that disinfection alone is insufficient to completely eliminate ARB and ARGs, highlighting the need to complement conventional treatments with advanced oxidation processes (AOPs) [15,17]. AOPs generate reactive oxygen species capable of oxidizing persistent organic compounds to complete mineralization, acting non-selectively through mechanisms such as hydrogen abstraction, radical addition, and electron transfer [18,19]. Among the most commonly used oxidants are ozone, ultraviolet (UV) light, and hydrogen peroxide [15]. AOPs are classified into four main categories—ozone-based processes, photocatalytic processes, ionizing radiation, and electron beam processes—as well as processes based on the Fenton reaction [19]. Among these, the photo-Fenton process has attracted particular interest in recent years due to its effectiveness in removing resistant bacteria and their associated genes, establishing itself as a promising strategy to overcome the limitations of conventional treatments [20].

The aim of this systematic review is to synthesize current evidence on the occurrence and risks of antibiotic resistance genes in wastewater, identify factors promoting horizontal gene transfer in WWTPs, and compare the effectiveness of conventional and advanced oxidation processes, with particular emphasis on photo-Fenton treatment.

## 2. Materials and Methods

This systematic review was conducted in accordance with the PRISMA 2020 (Preferred Reporting Items for Systematic Reviews and Meta-Analyses) statement. This methodology minimizes bias during the search and inclusion stages, thereby facilitating retrieval of high-quality publications directly relevant to the study objectives. This systematic review was registered in Open Science Framework (https://osf.io/qn3yc accessed on 1 May 2026). No amendments were made to the registration or protocol after commencement. If amendments are made, they will be documented and reported in the final manuscript.

### 2.1. Search Strategy and Information Sources

A comprehensive and systematic literature search was conducted across four major electronic databases: PubMed, ScienceDirect, Multidisciplinary Digital Publishing Institute (MDPI), and SpringerLink. The objective of this search was to identify relevant peer-reviewed publications reflecting the most recent advances in the field. To ensure the review emphasized contemporary evidence, only studies published within the last ten years (January 2015 through September 2025) were included. This decade represents a critical period of transition where molecular detection techniques, such as high-throughput quantitative PCR (qPCR) and Next-Generation Sequencing (NGS), reached standardized maturity, allowing for more precise quantification of resistance determinants compared to older studies. Furthermore, while the fundamental principles of advanced oxidation processes (AOPs) were established in earlier decades, the specific application of solar photo-Fenton at near-neutral pH and its optimization for the degradation of genetic material in complex wastewater matrices is a relatively recent research frontier. Therefore, this timeframe prioritizes contemporary data that reflect current global antimicrobial resistance (AMR) trends and the latest engineering advancements in sustainable oxidation processes.

The conceptual framework of the search is summarized in Table 1. The search strategy was intentionally focused on the specific combination of “ARGs,” “photo-Fenton,” and “wastewater” to maximize precision. To ensure reproducibility and transparency, the exact search strings provided.

### 2.2. Eligibility Criteria

Inclusion criteria. Studies were included if they met the following criteria: (i) focused on the occurrence, removal, or degradation of antibiotic resistance genes (ARGs) or antibiotic-resistant bacteria (ARB) specifically in wastewater matrices; (ii) evaluated advanced oxidation processes (AOPs), with emphasis on photo-Fenton; and (iii) provided quantitative data on removal efficiencies or kinetic parameters.

Exclusion criteria. The following types of studies were excluded: Studies focused solely on conventional biological treatment without tertiary or advanced oxidation stages. Studies without explicit data on ARG quantification (e.g., only chemical oxygen demand or general coliform counts). Individual case reports, books, technical manuals, editorials, book chapters, and publications without available full text. Non-peer-reviewed reports or studies published in languages other than English or Spanish.

### 2.3. Data Collection Process

**Figure 2:**
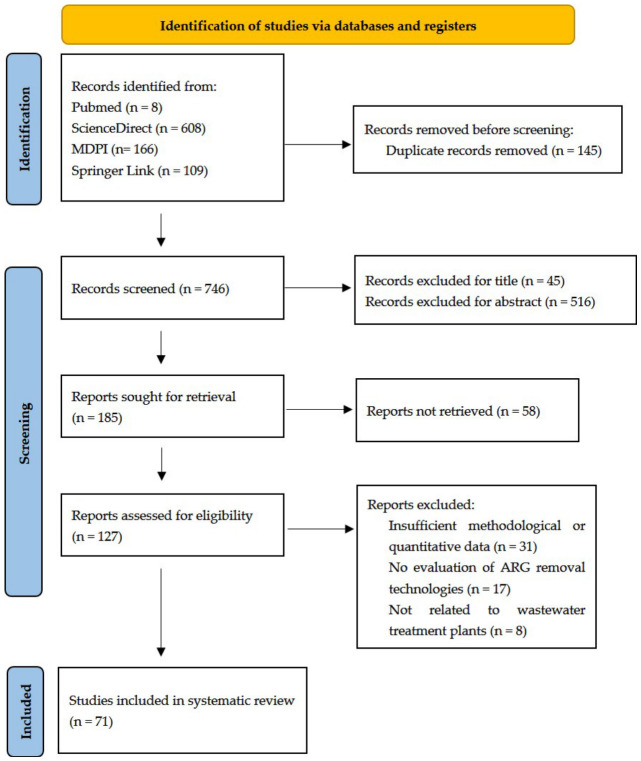
PRISMA 2020 flow diagram of the systematic literature review process.

The study selection and data collection process were conducted using the Rayyan platform (https://www.rayyan.ai/, accessed on 1 May 2026) to ensure a systematic, transparent, and reproducible workflow. All retrieved records were imported into the platform, where duplicate articles were automatically identified and removed. Subsequently, a two-stage screening process was performed. First, titles were reviewed, and studies not directly related to the scope of antibiotic resistance genes (ARGs) in wastewater were excluded. Second, abstracts were assessed to further refine the selection based on relevance to the study objectives. The remaining articles were evaluated through full-text screening. At this stage, studies were excluded based on predefined eligibility criteria, and the reasons for exclusion were documented. The entire selection process, including the number of records at each stage and exclusion reasons, is presented in Figure 2. After screening the titles and abstracts of the initially identified records, 185 papers were selected for full-text assessment. Of these, 58 reports were not retrieved. During the eligibility phase, 56 reports were excluded after full-text review because they did not specifically focus on the removal or degradation of ARGs, focusing instead on general bacterial counts or other contaminants. Consequently, 71 studies were included in the final systematic synthesis. Two independent reviewers screened the titles and abstracts of all retrieved records. Potentially relevant articles were then assessed in full text by the same two reviewers (M.R.D.-M. and J.A.R.-M.). Disagreements at any stage were resolved through discussion; if consensus could not be reached, a third reviewer (A.A.-R.) made the final decision.

### 2.4. Data Items

Data extraction was performed from all studies that met the inclusion criteria. The following variables were systematically collected:

- Bibliographic information (authors, year of publication);
- Study context and type of wastewater matrix;
- Target antibiotic resistance genes (ARGs);
- Type of treatment technology evaluated;
- Operational parameters (e.g., pH, oxidant concentration, irradiation time);
- Reported removal efficiencies (e.g., log reduction, percentage removal);
- Key findings related to ARG occurrence and horizontal gene transfer (HGT).

When information was incomplete or unclear, it was interpreted based on the context provided in the study.

### 2.5. Risk of Bias Assessment

The risk of bias of the included studies was qualitatively assessed based on methodological transparency and experimental robustness.

Each study was evaluated considering:

- Clarity and reproducibility of the experimental design;
- Adequacy of analytical and detection methods;
- Completeness of reported data;
- Consistency between objectives, methods, and results.

Based on these criteria, studies were categorized as having low, moderate, or high risk of bias.

### 2.6. Effects Measures

The primary effect measures used to evaluate the performance of treatment technologies included:

- Logarithmic reduction in ARGs;
- Percentage removal efficiency;
- Changes in ARG abundance.

These metrics were used to compare the effectiveness of conventional and advanced treatment processes across studies.

### 2.7. Synthesis Methods

A qualitative synthesis was conducted due to the heterogeneity of study designs, experimental conditions, and reported outcomes.

Studies were grouped according to:

- Type of treatment technology (conventional vs. advanced oxidation processes);
- Type of ARGs evaluated;
- Operational conditions and environmental matrices.

The synthesis focused on identifying patterns, comparing treatment efficiencies, and highlighting the advantages and limitations of each approach.

### 2.8. Reporting Bias Assessment

Potential reporting bias was assessed qualitatively by examining discrepancies between study objectives, methodologies, and reported results, as well as the selective reporting of outcomes. Particular attention was given to the absence of negative or non-significant findings, which may influence the interpretation of treatment effectiveness.

### 2.9. Certainty Assessment

The overall certainty of the evidence was evaluated qualitatively by considering:

- Consistency of findings across studies;
- Methodological quality and risk of bias;
- Reproducibility of reported results.

The body of evidence was interpreted with caution due to variability in experimental conditions and analytical approaches among studies.

### 2.10. Quality Assessment of Included Studies

A custom quality assessment was developed to evaluate the methodological rigor of the included studies, aligned with the reviewer’s proposed criteria. Rather than applying a pre-existing tailored tool, we assessed each study based on five key parameters: (i) matrix type (real vs. synthetic wastewater), (ii) experimental replication (*n* ≥ 3), (iii) reporting of PCR inhibitor control, (iv) ARG detection method, and (v) identification of key limitations. Based on these parameters, each study was assigned a quality score of High, Medium, or Low.

Table 2 summarizes the 71 studies included in this systematic review, presenting essential information such as the year of publication, article title, and study ID details. The selected studies span the period from January 2015 to September 2025, reflecting the rapid growth of scientific research addressing the environmental dimension of antimicrobial resistance, particularly the occurrence and control of antibiotic resistance genes (ARGs) in wastewater systems. The reviewed literature consistently identifies wastewater treatment plants (WWTPs) as critical reservoirs and dissemination pathways for antibiotic-resistant bacteria (ARB) and ARGs. Several studies investigate the environmental occurrence, persistence, and mobility of ARGs, highlighting the importance of horizontal gene transfer (HGT) as a key mechanism facilitating the spread of resistance within microbial communities in wastewater matrices. The presence of antibiotics, pharmaceuticals, heavy metals, and other contaminants at sub-inhibitory concentrations is frequently reported as a factor that can promote the proliferation and transfer of resistance determinants.

A considerable proportion of the studies focus on evaluating the effectiveness of treatment and disinfection technologies for the removal or inactivation of ARGs. Conventional methods such as chlorination, ozonation, and ultraviolet (UV) irradiation are widely investigated; however, the literature indicates that while these processes are effective in reducing bacterial populations, their ability to completely degrade extracellular genetic material is often limited. As a result, increasing research attention has been directed toward advanced oxidation processes (AOPs), including photo-Fenton, electro-Fenton, and other oxidative technologies, which have demonstrated promising potential for degrading genetic material through the generation of highly reactive oxidative species.

A temporal analysis of the selected studies reveals a progressive increase in research output after 2018, coinciding with the growing recognition of antimicrobial resistance as a global environmental and public health challenge. In addition, recent studies increasingly emphasize advanced treatment technologies and hybrid processes, highlighting the scientific community’s efforts to develop more efficient and sustainable strategies to mitigate the dissemination of ARGs into natural aquatic environments. Overall, the studies summarized in Table 2 demonstrate the global relevance of ARG contamination in wastewater systems and emphasize the urgent need for improved treatment approaches capable of limiting the environmental spread of antimicrobial resistance and its associated ecological and public health risks.

## 3. Results and Discussion

To ensure a rigorous interpretation of the findings, it is essential to distinguish between the various levels of treatment efficiency. In this study, bacterial inactivation refers to the loss of microbial viability or culturability (e.g., measured in CFU/mL), which does not necessarily imply the disappearance of genetic material. ARG removal is defined as the decrease in the detectable concentration of specific genetic sequences, typically measured via qPCR. However, this must be further distinguished from ARG degradation, which involves the physical fragmentation or mineralization of the DNA molecule to a degree that prevents its biological function. Finally, the reduction in transfer potential refers to the effective mitigation of horizontal gene transfer (HGT) risk, ensuring that remaining DNA fragments are no longer capable of transforming competent cells.

### 3.1. Occurrence of Antibiotic Resistance Genes (ARGs)

Sewer systems not only collect domestic wastewater but also receive effluents from other sources, such as industrial and hospital discharges. The latter are particularly relevant due to the toxic and infectious characteristics of their effluents, making them a significant source of ARGs and ARB [8]. Antibiotics are among the most widely administered drugs in medicine and therefore frequently reach aquatic environments, promoting the emergence of ARB and the dissemination of ARGs, which pose a risk to human health [21]. Moreover, antibiotics are not fully metabolized by humans and animals; approximately 85% are excreted in feces or urine, making them common components of wastewater. This explains the high concentrations of ARGs and ARB found in wastewater treatment plants [10,22]. In fact, it has been observed that ARG concentrations reach their highest levels during autumn and winter, coinciding with increased antibiotic prescriptions during these seasons. However, both ARB and ARGs remain poorly studied and lack monitoring in domestic wastewater [23].

Consequently, the indiscriminate use of antibiotics has contributed to the global rise in resistant bacteria. As a result, infections caused by these resistant bacteria do not respond effectively to conventional treatments, and are responsible for more than 700,000 human deaths annually [24]. It is estimated that, if this trend continues, antimicrobial resistance-related deaths could reach 10 million by 2050 [25]. Wastewater bacteria can develop antibiotic resistance intrinsically through various mechanisms. These mechanisms include reduced membrane permeability, biofilm formation, efflux pump activity, and gene expression, with the latter being the most critical factor [26].

**Figure 3:**
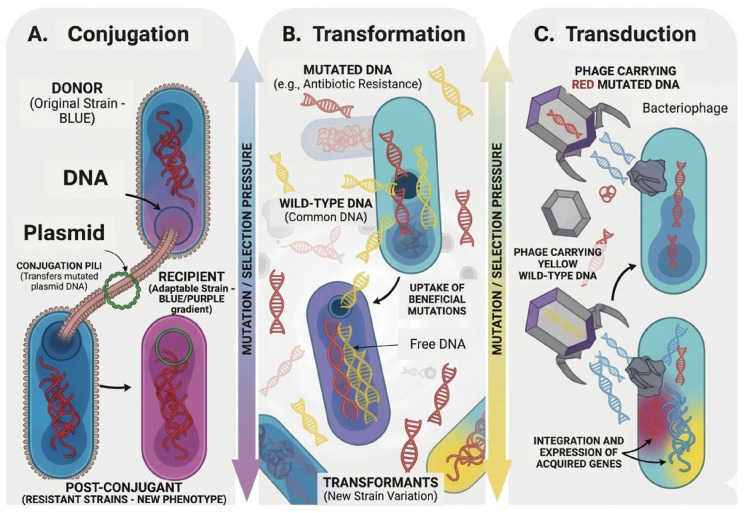
Mechanisms of horizontal gene transfer, (**A**) conjugation, (**B**) transformation, and (**C**) transduction.

Additionally, water harbors a vast diversity of microorganisms that can exchange ARGs through horizontal gene transfer (HGT) mechanisms. These include plasmids, transposons, and integrons, which facilitate the acquisition and dissemination of genetic material [27]. Such elements can carry resistance genes against different antibiotic families, which can in turn be shared between bacteria native to aquatic environments and pathogenic species [28]. Therefore, ARGs are considered emerging contaminants and represent one of the major global health threats [29]. In this context, horizontal gene transfer among bacteria can occur via three main mechanisms, as illustrated in Figure 3: conjugation, where a plasmid is directly transferred from a donor to a recipient bacterium through cell-to-cell contact; transformation, which involves the uptake of free DNA from the environment by bacteria and its incorporation into their chromosome or a plasmid; and transduction, in which a bacteriophage carries DNA from one bacterium to another [30,31].

In recent years, several studies have investigated the occurrence and identification of ARGs, ARB, and mobile genetic elements associated with bacterial resistance (Table 3). These studies have covered hospital wastewater, influents and effluents from WWTPs, as well as water bodies such as rivers and lakes [28]. Various ARGs belong to multiple antibiotic families, including aminoglycosides, β-lactams, macrolides, quinolones, sulfonamides, and tetracyclines, and have been frequently detected in water and soil [25,32].

The systematic synthesis presented in Table 3 reveals a clear disparity in the environmental persistence of ARGs. Specifically, *sul*1 and *sul*2 consistently exhibit the highest abundance in effluents and the lowest removal rates by conventional activated sludge processes. This persistence is strongly linked to their association with Class 1 integrons, which facilitate their stabilization and horizontal dissemination even in the absence of high antibiotic selective pressure. In contrast, while β-lactam genes are frequently detected, they show higher susceptibility to conventional disinfection, although their presence in the extracellular DNA fraction remains a critical concern for downstream environmental integrity. Sulfonamides and β-lactams represent the highest relative proportions of antibiotics detected in urban effluents, frequently reaching concentrations in the µg/L range. These proportions are critical for wastewater treatment design, as high concentrations of sulfonamides like SMX have been shown to correlate with the increased prevalence of *sul*1 and *sul*2 genes. Furthermore, the presence of these compounds at sub-inhibitory levels acts as a primary driver for the horizontal gene transfer (HGT) discussed in Section 3.2. Understanding these relative abundances is essential when optimizing advanced oxidation processes (AOPs), as the chemical structure of each antibiotic class determines its specific degradation kinetics during the photo-Fenton reaction. In this context, ARGs present in effluents, such as *sul*1, *sul*2, and *erm*B, stand out as high-risk genes due to factors including their origin, genetic mobility, host pathogenicity, and expression potential, highlighting their significant impact on public health [35].

### 3.2. Conditions Favoring Horizontal Gene Transfer

**Figure 4:**
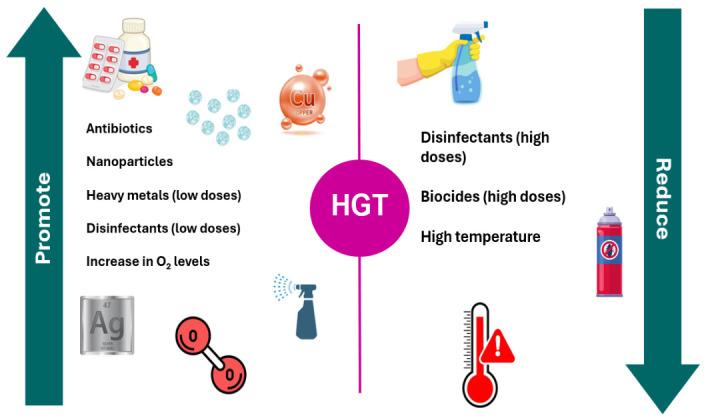
Factors that promote and mitigate horizontal gene transfer. Adapted from [31].

Within WWTPs, the presence of antibiotics, metals, and other chemical compounds can generate selective pressure that promotes HGT [36], as depicted in Figure 4. Exposure to antibiotics, even at low concentrations, can accelerate this transfer in environmental samples due to their ability to select for resistant bacteria [31]. For example, a tetracycline concentration as low as 10 μg/L (equivalent to 150 times lower than the minimum inhibitory concentration) increases HGT in activated sludge and WWTP effluents [37]. Beyond antibiotics, several pharmaceuticals, including ibuprofen, naproxen, diclofenac, and propranolol, have been shown to increase transformation rates by 1.9 to 2.4 times [14]. Similarly, other conditions, such as chlorination at subinhibitory concentrations (0.1–1 mg Cl_2_/L), have been reported to enhance horizontal gene transfer by 1.9 to 7.5 times [38].

A recurring finding in the analyzed studies is that WWTPs act as reservoirs where HGT is drastically accelerated [3,27]. Regarding selective pressure: the presence of contaminants at sub-inhibitory concentrations (ng/L–μg/L) in bioreactors is the main driver of selection [15]. Authors such as Jutkina et al. [37] have shown that tetracycline concentrations 150 times lower than the minimum inhibitory concentration already increase gene transfer in activated sludge. Concerning co-contaminants: the review highlights that not only antibiotics promote resistance; commonly used pharmaceuticals such as ibuprofen and diclofenac can increase transformation rates up to 2.4 times [14]. Likewise, chemical elements such as Fe^3+^ ions and humic acid play a dual role, being able to partially mitigate the risk of dissemination from high-risk effluents [14].

The presence of heavy metal ions, such as iron (Fe^2+^/Fe^3+^), silver (Ag^+^), and copper (Cu^2+^), at sub-lethal concentrations acts as a powerful catalyst for the dissemination of multi-resistance. The primary mechanism involves the induction of oxidative stress, which triggers the bacterial SOS response. This global regulatory network increases the expression of genes involved in DNA repair and, crucially, promotes the mobilization of mobile genetic elements (MGEs) such as integrons and plasmids. Furthermore, metal ions can enhance cell membrane permeability by altering the lipid bilayer integrity, physically facilitating the uptake of extracellular DNA (eDNA) through natural transformation. Specifically, Cu^2+^ has been shown to upregulate the expression of *tra* genes, which are essential for the formation of the mating bridge during conjugation, thereby increasing the transfer frequency of multi-resistance plasmids among diverse bacterial taxa.

Moreover, oxygen levels influence bacterial community composition and the proliferation of ARGs; aerobic sludge contains more Proteobacteria and up to twice, the abundance of plasmids compared to anaerobic sludge [39]. In addition, the presence of nanoplastics significantly increases the horizontal transfer of extracellular antibiotic resistance genes (eARGs), thereby raising the risk of antibiotic resistance dissemination. However, it has been shown that the presence of humic acid or Fe^3+^ ions can partially mitigate this effect [14]. Likewise, both CuO nanoparticles and Cu^2+^ ions have been shown to promote the conjugative transfer of ARGs, even at subinhibitory concentrations and under environmentally relevant conditions [40]. Similarly, subinhibitory levels of both silver nanoparticles and silver ions have been reported to facilitate the transfer of multiple resistance genes from *Escherichia coli* K-12 LE392 to *Pseudomonas aeruginosa* KT2440, thereby enhancing the spread of AMR [41].

Ultimately, biological treatment processes can increase the abundance of certain ARGs, leading to higher concentrations in the effluent than in the influent. Therefore, it is essential to implement more effective treatment methods to reduce the presence of ARGs in WWTP effluents [41].

Our quality assessment (Table 4) reveals a significant disparity in the reporting of molecular protocols. Approximately 40% of the reviewed studies failed to report the use of internal controls for PCR inhibition, which is critical when dealing with complex matrices like hospital wastewater. Consequently, the reported “high removal” rates in these studies must be interpreted with caution, as they may reflect analytical interference rather than true genetic degradation. Conversely, studies that employed standardized protocols provide a more reliable basis for comparing the effectiveness of advanced oxidation processes.

### 3.3. Risks of ARGs in WWTPs and Their Impact on Human Health and the Environment

**Figure 5:**
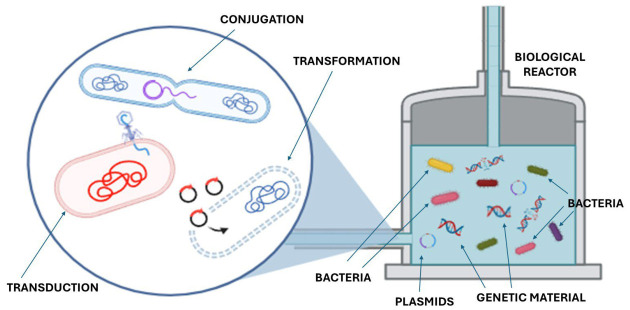
Schematic representation of horizontal gene transfer during secondary treatment.

Within WWTP facilities, four main treatment stages can be distinguished: pre-treatment, aimed at removing large solids; primary treatment, which removes suspended solids and partially reduces organic matter; and secondary treatment, in which organic matter is removed through biological processes [42]. It is precisely during this stage that ARG transfer is promoted (Figure 5), as conditions are favorable for bacterial growth and increased selective pressure enhances the proliferation of ARGs [43]. Finally, tertiary treatment disinfects the effluent to prevent the spread of pathogens [42]. Consequently, the presence and abundance of ARGs in WWTPs vary depending on the treatment processes implemented and the efficiency of the individual treatment units [10].

For instance, during primary treatment, ARG removal is relatively ineffective, with decreases ranging from 0.09 to 0.55 log units. In contrast, secondary and tertiary treatments achieve more substantial reductions, typically between one and two orders of magnitude. Additionally, a portion of ARGs and ARB is removed in the aqueous phase, while another fraction accumulates in sludge. Nevertheless, a proportion of these genes persists in the treated effluent, posing a risk of contact with environmental bacteria, animals, and humans [10,31]. In this context, ARG hosts can transfer these genes to commensal and pathogenic bacteria via horizontal gene transfer. For example, the *bla*CTX-M genes and quinolone resistance genes originated from the chromosomes of *Kluyvera* and *Shewanella*, respectively, and have since spread globally through plasmids [31].

In addition, ARGs present in treated wastewater can lead to soil contamination when such water is used for agricultural irrigation. These genes can spread through soil bacteria via HGT, potentially affecting crops and the food supply [43]. Furthermore, sludge accumulation in WWTPs promotes high microbial density and facilitates horizontal gene transfer, which may result in resistance to multiple antibiotics. From this perspective, sludge represents a reservoir of ARGs that can be reintroduced into the system or released into the environment when applied as landfill material or fertilizer, posing a potential risk to human and animal health [10].

Therefore, growing concern over bacterial resistance has driven the development of new antibiotics as well as the adoption of strategies aimed at limiting its spread. This requires improvements in wastewater treatment processes, as WWTPs represent one of the main vectors for resistance dissemination [44]. In this context, conventional disinfection processes eliminate bacteria through different mechanisms, and operational conditions can influence the extent of cellular damage and, consequently, the ability of affected cells to repair and reproduce [45]. However, eliminating ARB and preventing their proliferation is not sufficient to combat antimicrobial resistance; it is equally essential to degrade both intracellular and extracellular ARGs [42].

### 3.4. Conventional Methods for the Removal of Antibiotic Resistance Genes

#### 3.4.1. Chlorination

Chlorine is considered one of the most widely used disinfectants due to its broad-spectrum activity against various microorganisms, high effectiveness, and low cost [46]. Given the relevance of environmental and public health risks associated with the presence of microorganisms, chlorination has proven to be a commonly used disinfection method in water treatment and sanitation, being effective in controlling the spread of pathogens [47]. Chlorine exerts its disinfecting function through the action of hypochlorite and hypochlorous ions, which irreversibly oxidize proteins, resulting in enzyme inactivation and cell death [5].

However, chlorination can increase cell membrane permeability, thereby facilitating the horizontal transfer of ARGs. Consequently, some microorganisms, particularly those pathogenic to humans, pose a threat to human health and the environment [47]. Moreover, this process may increase the abundance of both intracellular and extracellular ARGs in wastewater, thus contributing to the dissemination of antimicrobial resistance [46]. Therefore, some studies have evaluated the effect of chlorine on extracellular ARGs (eARGs), revealing a biphasic behavior during disinfection: an initial increase due to bacterial inactivation and the release of eARGs into the medium, followed by a decrease caused by oxidative degradation [14].

It has been demonstrated that only high concentrations of chlorinated agents (30 mg/L) can significantly reduce the abundance of ARGs. However, excessive use has been associated with the formation of disinfection by-products, which in turn increases the dissemination of ARGs and ARB [48]. In addition, chlorination acts selectively on eARGs, favoring those with high antioxidant capacity, such as the *amp*C gene, while eliminating genes with lower antioxidant capacity, such as *amp*R. This behavior highlights the complexity of eARG responses to chlorination processes [14].

#### 3.4.2. Ozonation

Ozone is a strong oxidizing agent and has been increasingly used in wastewater treatment in recent years, as it reacts specifically with organic compounds through two pathways: a direct reaction with ozone molecules and an indirect reaction involving •OH radicals [49]. It has been demonstrated that the effectiveness of ozonation disinfection depends on water quality, contact time, and the applied ozone dose. For instance, the application of 1 g of ozone per gram of dissolved organic carbon achieved significant reductions in the resistance genes *TEM*, *erm*B, *sul*, and *bla* of 98%, 95%, 91%, and 91%, respectively [50]. Nevertheless, ozonation is a costly disinfection method, lacks residual effects, and may lead to the formation of toxic by-products [51]. Although several studies have demonstrated the high efficiency of ozonation in ARG removal, it has also been reported that this process does not prevent the potential regrowth of resistant bacteria and resistance genes [25].

Furthermore, very high ozone doses are required to achieve penetration into the bacterial cytoplasm and the destruction of ARGs, which may result in the formation of disinfection by-products [48]. Regarding eARGs, ozonation has shown an efficiency of up to 90% under controlled conditions; however, in WWTPs its effectiveness decreases due to the complexity of the wastewater matrix [14]. In addition, the effectiveness of ozone disinfection can be compromised by high concentrations of radical scavengers, such as carbonates, halogens, or nitrogen oxides. Moreover, the presence of halogens may lead to the formation of toxic oxidation by-products [52].

#### 3.4.3. Ultraviolet Radiation

Ultraviolet (UV) radiation directly damages microbial DNA, leading to cell death or inhibition of reproduction, while simultaneously inducing photochemical damage to nucleic acids, which makes it effective for the removal of ARGs [29,41]. However, exposure to low doses of UV radiation may increase the abundance of ARGs, particularly those associated with efflux pumps. This effect is further amplified after a second exposure as a result of plasmid replication and horizontal gene transfer among bacteria [41].

With respect to eARGs, exposure to UV light reduces their abundance by damaging their structure, thereby limiting their dissemination. At the same time, the degradation efficiency of these genes depends on factors such as gene length and structure, the presence of active sites along the DNA chain, and the intensity of the applied UV radiation [14]. Consequently, several studies indicate that UV radiation acts selectively on ARGs and that its capacity to degrade DNA or genetic fragments is limited [52]. Although UV radiation offers advantages such as being non-corrosive and not generating chemical residues, it does not provide continuous disinfection due to the phenomenon of photoreactivation, which may occur after treatment. In addition, very high doses are required to achieve optimal effectiveness [10].

Accordingly, it has been estimated that effective ARG inactivation requires UV radiation doses 10 to 100 times higher than those commonly applied in wastewater treatment (<100 mJ/cm^2^). Nevertheless, the high energy demand limits its practical application [48]. Although conventional disinfection processes (chlorination, UV irradiation, and ozonation) are effective in eliminating ARB, most ARGs can persist extracellularly [53]. This limitation highlights the need to explore alternative strategies capable of effectively removing ARGs. Currently, many WWTPs lack the proper equipment to address the challenges of ARB and ARGs removal and, in general, do not include oxidative treatment stages in their processes [44]. In this context, wastewater treatment technologies targeting ARB and ARGs can be classified into two main approaches: (1) removal through capture and retention, and (2) degradation of cellular integrity via reactive oxygen species [54].

### 3.5. Advanced Oxidation Processes

AOPs are characterized by the generation of •OH radicals, which are highly reactive species with a high oxidation potential (E° = 2.8 V vs. Standard Hydrogen Electrode), capable of degrading a wide range of contaminants [51]. Moreover, AOPs are effective methods for bacterial inactivation and ARG removal, as the produced free radicals cause damage to both the cell membrane and the DNA structure [55].

To evaluate the practical applicability of different treatment trains, it is essential to compare conventional removal methods with advanced oxidation processes (AOPs). While traditional methods focus on physical separation or biological reduction, AOPs aim for molecular destruction.

The primary advantage of AOP-based methods, particularly photo-Fenton, lies in their ability to achieve mineralization rather than simple sequestration. Unlike membrane processes that merely transfer the resistance load to a concentrate, AOPs attack the phosphodiester bonds of the DNA backbone. However, a significant disadvantage is the “scavenging effect” where natural organic matter (NOM) and carbonates compete for hydroxyl radicals, potentially reducing the efficiency of ARG fragmentation in complex real-world effluents.

#### 3.5.1. Photocatalytic Oxidation

H_2_O_2_ is a strongly oxidizing agent and is also environmentally friendly, as it degrades into oxygen and water without producing harmful by-products. However, its disinfectant capacity depends on the initial concentration, and it has been suggested to enhance its action through activation with other oxidants or catalysts to increase treatment efficacy [24]. Several laboratory-scale studies have shown that photocatalytic oxidation is highly effective for ARG removal. For instance, a UV/H_2_O_2_ process operated at pH 3.5, with an H_2_O_2_ concentration of 0.01 mol/L and 30 min of UV irradiation, achieved a reduction in ARGs between 2.8 and 3.5 log units [56].

Nevertheless, in studies conducted with real wastewater samples, the UV/H_2_O_2_ process applied for 60 min did not achieve complete ARG removal (*bla*TEM, *qnr*S, and *tet*W), yielding only a reduction in intracellular ARGs. This low efficiency was attributed to the limited oxidant concentration, the weak action of •OH on DNA, and its rapid neutralization by compounds present within the cells [10]. A similar study reported that although the UV/H_2_O_2_ process achieved a significant reduction in ARGs, it failed to completely eliminate the *bla*TEM gene from total DNA even after 240 min of treatment [57]. It has been reported that removal efficiency improves with increased H_2_O_2_ concentration; however, excessive amounts of this compound can slow down the process due to secondary reactions that regenerate H_2_O_2_, thereby reducing its overall efficacy [10].

Over the past decades, heterogeneous photocatalytic processes have emerged as an efficient alternative for water disinfection by promoting the formation of reactive oxygen species through the irradiation of a semiconductor, such as TiO_2_, with ultraviolet light [24]. In this context, TiO_2_ photocatalysis has demonstrated its effectiveness in ARG removal, achieving reductions in mecA and ampC genes by 5.8 and 4.7 log units, respectively, under a UV radiation dose of 120 mJ/cm^2^, eliminating both intracellular and extracellular ARGs [58]. Furthermore, Gajdoš et al. (2023) employed graphene oxide combined with TiO_2_ and observed a decrease in ARGs; however, the DNA concentration remained constant due to its release without complete degradation [59]. Nonetheless, photocatalytic oxidation presents limitations, such as low light utilization efficiency, potential inactivation and release of the TiO_2_ catalyst into the environment, and higher costs compared to conventional methods [56].

#### 3.5.2. Electrochemical Oxidation

**Figure 6:**
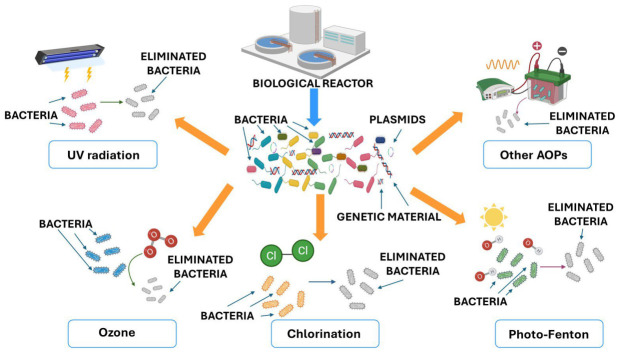
Methods for antibiotic resistance genes removal in effluents.

Electrochemical disinfection is an alternative for the removal of resistant bacteria and their genes, notable for its high efficiency, low energy consumption, reduced environmental impact, and simple operation [25]. This technology can be implemented without the need for chemical storage, unlike methods such as chlorination, ozonation, or the Fenton process, which reduces associated operational costs [24]. In electrochemical disinfection, cells are primarily inactivated by damage to the cell membrane, caused both by the direct current and by oxidants generated at the anode, such as ozone, oxygen radicals, and reactive chlorine species [60]. Additionally, the efficiency of electrochemical disinfection depends on the electrode material, electrolyte composition, and operational conditions, such as current density [24]. Recent studies demonstrate that electrochemical disinfection can simultaneously remove ARGs and ARB. For instance, Fang et al. (2022) achieved a 5 log reduction in *Escherichia coli* K-12 LE392 and degradation of the *bla*TEM and *tet*A genes using a molybdenum carbide-assisted system at 2 V [61]. However, it has been observed that sublethal electrochemical conditions can increase the transfer of plasmids carrying ARGs, as chlorine radicals and superoxide generate oxidative stress, stimulating the expression of genes related to conjugation [60]. Figure 6 outlines different methods for ARG removal in effluents.

#### 3.5.3. Fundamentals of the Photo-Fenton Process

In 1894, Henry J. Fenton first described the Fenton process, observing that hydrogen peroxide was capable of oxidizing tartaric acid when activated by ferrous ions [62]. Thus, the Fenton process consists of a reaction in an acidic medium between Fe^2+^ and H_2_O_2_, known as Fenton reagents, whose purpose is to generate •OH radicals (Equation (1)), capable of non-selectively degrading organic matter [34,63].

Fe^2+^ + H_2_O_2_ → Fe^3+^ + OH^−^ + •OH (1)

**Figure 7:**
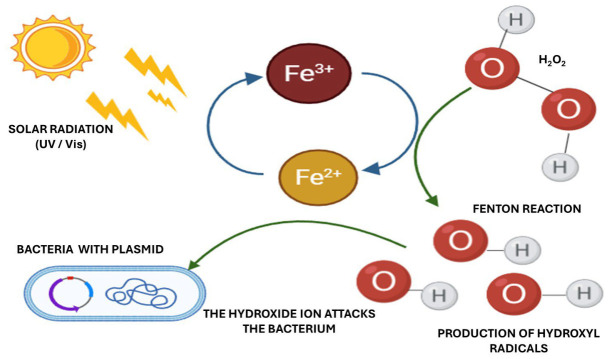
Mechanism of the photo-Fenton process.

The reactions of the Fenton process involve the oxidation of Fe^2+^ to Fe^3+^ along with the production of •OH and the reduction in Fe^3+^ to Fe^2+^ (Figure 7). However, the reaction rate of oxidation is 6000 times faster than reduction, preventing an efficient iron regeneration cycle and leading to Fe^3+^ accumulation. At a pH above 3, Fe^3+^ begins to precipitate as iron oxyhydroxide, commonly called iron sludge [64].

Some advantages of the Fenton process include its ability to degrade recalcitrant contaminants at room temperature and atmospheric pressure in short times, as well as the easy access and low cost of the reagents. However, its major limitation is the optimal working pH range, which is around 2.8 [34,65]. The Fenton process can be optimized by combining it with UV irradiation, resulting in the so-called photo-Fenton process, which significantly increases •OH generation, both through the Fenton reaction and the action of UV light on the iron complex (Fe(OH)^2+^) formed during the process [24]. Therefore, the photo-Fenton process can be defined as the generation of •OH from H_2_O_2_ using iron salts (Fe^2+^ and Fe^3+^) and UV light as a catalyst [66].

However, using UV radiation during the photo-Fenton process can increase operational costs, which has led to the alternative of employing sunlight as a renewable energy source [24]. Consequently, the photo-Fenton process presents an efficient and cost-effective option for removing ARB and their resistance genes [25]. Nevertheless, the main limitation of the photo-Fenton process is the requirement for an acidic pH to prevent iron precipitation. The use of chelating agents allows stability and operation at near-neutral pH [44,67].

Among the most commonly used chelating agents to maximize the photo-Fenton process are ethylenediaminetetraacetic acid (EDTA), which is non-biodegradable; nitrilotriacetic acid (NTA), which is potentially carcinogenic; and ethylenediamine disuccinic acid (EDDS), whose main drawback is its high cost. Additionally, some of these agents do not have the capacity to remove ARGs [67]. Besides pH, other operational parameters, such as reagent concentration, matrix complexity, and radiation intensity, can influence process efficiency [42]. In particular, irradiance can vary depending on factors such as weather, latitude, and solar radiation intensity, which represents a challenge for maintaining reactor efficiency and properly controlling the process [52].

**Figure 8:**
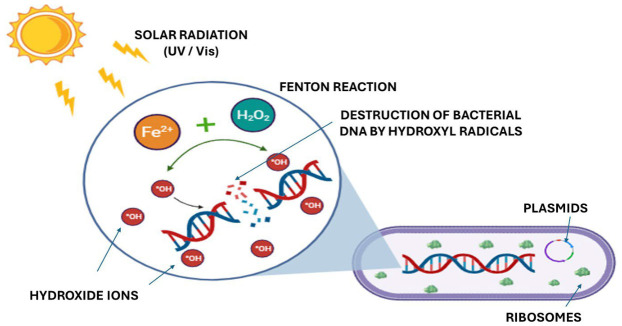
Attack of hydroxyl radicals produced in the photo-Fenton process on the genetic material of antibiotic resistance bacteria.

Since H_2_O_2_ and Fe^2+^ can be present inside and outside bacterial cells and penetrate their cell walls, the •OH radicals generated simultaneously attack the membrane and internal components such as DNA, lipids, and proteins (Figure 8). UV light, in turn, inhibits enzymes that remove intracellular H_2_O_2_, promoting the release of Fe^2+^ from iron-containing proteins, thereby increasing the Fenton reagents within the cell and favoring intracellular photo-Fenton reactions [44].

#### 3.5.4. Studies on ARG Removal by the Photo-Fenton Process

Several studies have confirmed that the photo-Fenton process effectively eliminates ARB, removes ARGs, and inactivates free ARGs [42,55]. The solar photo-Fenton process using iminodisuccinic acid as a chelating agent proved effective, achieving a 1.4 log reduction for *Staphylococcus* spp. and a 4.3 log reduction for *Escherichia coli*, with no significant regrowth observed. Regarding ARGs, removal efficiency varied depending on the gene: *qnr*S showed a marked reduction, whereas *gyr*A, *mef*A, and *int*I1 exhibited only minimal decreases [67].

In another study, the solar photo-Fenton process was evaluated at neutral pH (30 mg/L Fe^2+^ added intermittently and 50 mg/L H_2_O_2_) for the removal of the most common ARGs found in municipal WWTPs. Approximately 60% of ARGs associated with sulfonamides, macrolides, and tetracyclines were removed. In addition, complete elimination of ARGs associated with β-lactam antibiotics and fluoroquinolones was achieved [33]. Conversely, Fiorentino et al. [68] evaluated five genes: *sul*1, *qnr*, *bla*TEM, *bla*CTX-M, and *int*I1. Unlike bacterial and antibiotic removal, the effect of the solar photo-Fenton process on *int*I1, *bla*TEM, and *sul*1 was less pronounced. Variations in relative abundance before and after treatment depended on the specific ARG and the type of wastewater. The treatment was conducted using 50 mg/L H_2_O_2_ and 20 mg/L Fe^2+^, indicating that more intensive oxidation conditions are required to achieve higher removal efficiencies [68]. Michael et al. [34] demonstrated that the solar photo-Fenton process achieved complete ARB elimination after 60 min, also preventing bacterial regrowth after 24 h. Likewise, resistance genes *bla*OXA, *bla*CTX-M, *qnr*S, sul1, and *tet*M were reduced to the limit of quantification after 60 min of treatment, highlighting the effectiveness of the process [34].

The combination of the Fenton process with UV radiation reduced *tet*Q and *erm*B genes below the detection limit, unlike treatments based on ozonation or UV radiation alone. This high efficiency is attributed to the diffusion of Fenton reagents into the cytoplasm, triggering intracellular reactions in which •OH radicals cause severe DNA damage, irreversible mutations, and ultimately cell death. In contrast, the lower effectiveness of UV or ozone disinfection for ARG removal may be due to resistance mechanisms exhibited by host bacteria against these treatments [69]. It is worth noting that the susceptibility of ARGs to •OH radicals varies according to their composition, as this influences hydrophilicity, reactivity, and stability. For example, ARGs with high guanine and cytosine content, such as *sul*1 and *sul*2, are more readily degraded than tetracycline resistance genes, such as *tet*O, *tet*Q, and *tet*G [42]. Therefore, optimizing treatment conditions to target the most relevant ARGs in wastewater is of critical importance. Most studies have focused on the removal of ARGs associated with bacterial DNA. However, it is also essential to eliminate mobile genetic elements that transport extracellular ARGs.

In this context, the wastewater matrix itself acts as a protective medium, as total organic carbon can promote the abundance and persistence of eARGs by providing adsorption-based protection that prevents degradation by DNases [14]. Consequently, it has been demonstrated that the solar photo-Fenton process at neutral pH is effective in eliminating and inactivating antibiotic-resistant plasmids in both synthetic and real wastewater. Complete removal and inactivation were achieved within 30 min in secondary wastewater under sunlight, whereas 60 min were required in the absence of light [55]. Furthermore, the efficiency of homogeneous photo-Fenton (with dissolved iron in solution) was compared with that of heterogeneous photo-Fenton (with iron in solid form), revealing that the homogeneous process rapidly degraded a high percentage of iARGs within only 15 min. Notably, only this approach effectively reduced the concentration of the *bla*CTX-M-9 gene, indicating that ARGs, rather than resistant bacteria themselves, constitute the primary concern and that treatment systems should prioritize their degradation [44]. In another study, visible LED light at neutral pH achieved complete and permanent disinfection of *Escherichia coli* DH5α carrying the *tet*A and *bla*TEM-1 genes. Regarding eARGs, reductions between 6.8 and 8.6 log units were achieved. However, iARGs required higher reagent doses for degradation, with *tet*A being more susceptible than *bla*TEM-1 [70]. Finally, the removal of ARB through exposure to solar light and the solar photo-Fenton process was evaluated. Both methods proved effective, with no increase in resistance observed after treatment. In both cases, ARG reduction was achieved, although the solar photo-Fenton process was faster and more efficient [71].

The efficiency of the photo-Fenton process is highly sensitive to operational variables and the complexity of the aqueous matrix. To move beyond descriptive analysis, Table 5 synthesizes the key findings from the reviewed literature, highlighting the heterogeneity in pH levels, iron/peroxide dosages, and the differential removal rates for intracellular and extracellular ARGs.

Table 6 broadens the perspective by comparing the advantages and disadvantages of all ARG removal technologies reviewed, including chlorination, UV radiation, ozone, electrochemical oxidation, photocatalysis, classical Fenton, and photo-Fenton. Together, these tables show that while photo-Fenton is highly effective against both intracellular and extracellular ARGs, it shares with other AOPs common limitations such as pH dependency, reagent costs, and the need for optimized operating conditions.

Beyond technical performance, the economic feasibility of these methods is a decisive factor for their integration into municipal WWTPs, as shown in Table 7. Among the treatment methods reviewed for the removal of ARGs from municipal effluents, chlorination represents the low operational cost but low ARG removal efficiency. While chlorination benefits from widely available infrastructure and low chemical costs, achieving meaningful ARG degradation would require impractically high chemical consumption, limiting its large-scale adoption for ARG mitigation. In contrast, solar photo-Fenton presents a more sustainable economic profile. By leveraging natural sunlight and using relatively inexpensive catalysts (iron salts) and oxidants (H_2_O_2_), it reduces the electrical dependency associated with ozone or UV-based processes. However, the economic analysis must also account for hidden costs, such as the acidification/neutralization steps required for traditional Fenton reactions, which underscores the importance of researching neutral-pH chelating agents to further improve the cost–benefit ratio of AOPs.

### 3.6. Final Remarks

The analysis of the collected literature reveals a significant gap between traditional disinfection and genetic degradation. While conventional methods such as chlorination, ozonation, and UV radiation are effective for inactivating antibiotic-resistant bacteria (ARB), their capacity to eliminate persistent genetic material is insufficient [15,16]. Regarding the limitations of UV and chlorine, according to Li et al. (2017) [16], intact DNA can persist even after bacterial inactivation by disinfection. Furthermore, low doses of UV can paradoxically increase the abundance of ARGs through plasmid replication [41]. On the other hand, chlorination at sub-inhibitory concentrations can facilitate HGT by increasing cell membrane permeability [38]. Concerning the superiority of Photo-Fenton: In contrast to conventional photocatalysis (TiO_2_), which presents limitations in light utilization efficiency [56], the photo-Fenton process stands out for its capacity to efficiently generate •OH [18,19]. This technology overcomes the inherent limitations of conventional processes by achieving not only cell inactivation but also the definitive degradation of ARGs [20,42].

The accumulation of ARGs in treatment sludge represents a latent threat to public health. Regarding sludge as a reservoir: According to Pei et al. [10], a considerable fraction of ARGs is transferred to the solid phase during secondary treatment. This sludge, if used as fertilizer, reintroduces resistance genes into the soil, potentially affecting the food chain [10,43]. Concerning seasonal impact: It is relevant to observe that ARG concentrations reach maximum levels in autumn and winter, directly correlating with the increase in antibiotic prescriptions [23]. This seasonal pattern underscores the need for WWTPs to adjust the intensity of their treatments (especially tertiary and quaternary stages) according to the seasonal biological load.

Finally, the discussion converges on the fact that the legal vacuum is the main barrier to AMR control. The absence of standards establishing permissible limits for antibiotics and resistance determinants in treated effluents perpetuates the risk [44]. The combined implementation of degradation technologies, specifically the solar photo-Fenton process appears to be one of the most robust mechanisms for the substantial degradation of ARGs, potentially offering removal efficiencies that surpass those of conventional treatment methods. The photo-Fenton process and strict legislation are the only mechanisms capable of reversing this global threat [34,42].

## 4. Conclusions

A critical synthesis of the reviewed literature reveals significant heterogeneity in Photo-Fenton efficiency, largely driven by the water matrix and the physical state of the target DNA. While studies in synthetic water report high mineralization rates, results in real municipal effluents are often contradictory. This discrepancy is attributed to the “scavenging effect” of organic matter and carbonates, which compete for hydroxyl radicals. Furthermore, our analysis identifies a reporting bias; most studies focus on intracellular ARG removal—often directly linked to bacterial inactivation—while the more persistent extracellular ARG fraction remains underreported, potentially masking the true risk of horizontal gene transfer in treated effluents.

The threat of antimicrobial resistance is intensified in wastewater treatment plants as a result of the limited effectiveness of conventional treatment processes and the continuous selective pressure exerted by residual contaminants. A critical technological limitation lies in the inability of current treatment systems to effectively degrade resistance-associated DNA, thereby facilitating the persistence and dissemination of resistance determinants in the aquatic environment. Among advanced treatment alternatives, solar photo-Fenton processes emerge as a key and promising technology due to their demonstrated capacity to achieve the (irreversible removal) persistent reduction in the structural integrity of ARGs, both in their intracellular and extracellular forms. This capability represents a pivotal step toward limiting the environmental spread of AMR through treated effluents. Nevertheless, a comprehensive mitigation of this challenge cannot rely solely on technological solutions. The establishment of robust regulatory frameworks and legally binding guidelines defining permissible environmental thresholds for antibiotics and resistance determinants is imperative. The integration of stringent regulatory measures with efficient, sustainable, and advanced treatment technologies constitutes the most effective strategy to safeguard environmental integrity and public health against this escalating global threat.

While conventional wastewater treatment plants are not specifically designed for genetic material removal, the integration of advanced oxidation processes, particularly solar photo-Fenton, appears to be among the most promising approaches for addressing the persistence of ARGs in effluents. The evidence synthesized in this review supports photo-Fenton as a strong candidate for advanced tertiary treatment, as it facilitates the degradation of extracellular DNA that other methods may overlook. However, the term “removal” must be approached with caution, as scalability, energy costs, and complex matrix effects remain important challenges for full-scale implementation. While no single technology currently offers a universal solution for the total elimination of environmental ARGs, this review identifies solar photo-Fenton as the most suitable method for the tertiary treatment of ARGs in municipal effluents. This selection is based on its unique ability to achieve effective DNA mineralization rather than simple physical sequestration, significantly reducing the risk of horizontal gene transfer. Economically, the integration of solar radiation as a primary energy source provides a sustainable advantage over electricity-dependent processes like ozonation or membrane filtration, lowering operational costs in regions with high solar irradiance. However, for large-scale implementation to be successful, future research must prioritize the development of neutral-pH processes and the optimization of iron-recovery cycles to minimize sludge production. Ultimately, the transition from conventional disinfection to AOP-based tertiary treatments, supported by robust legislative frameworks, represents the most promising strategy to mitigate the global threat of antibiotic resistance dissemination from wastewater treatment plants.

## Figures and Tables

**Table 1:** Detailed search syntax by database for the retrieval of relevant records (January 2015 to September 2025).

Database	Search Strings	Articles Found
PubMed	(“ARGs” AND “Solar photo Fenton” AND “wastewater”) (“ARGs” AND “photo Fenton” AND “wastewater”)	8
ScienceDirect	(“ARGs” AND “Solar photo Fenton” AND “wastewater”) (“ARGs” AND “photo Fenton” AND “wastewater”)	608
Multidisciplinary Digital Publishing Institute (MDPI)	(“ARGs” AND “Solar photo Fenton” AND “wastewater”) (“ARGs” AND “photo Fenton” AND “wastewater”) (“ARGs” AND “wastewater”)	166
SpringerLink	(“ARGs” AND “Solar photo Fenton” AND “wastewater”) (“ARGs” AND “photo Fenton” AND “wastewater”)	109

**Table 2:** Studies selected for the systematic review.

Number	Year	Title	Internal Study ID *
1	2015	Occurrence of antibiotics and antibiotic resistance genes in hospital and urban wastewaters and their impact on the receiving river	Rodriguez-Mozaz et al., 2015
2	2016	An assay for determining minimal concentrations of antibiotics that drive horizontal transfer of resistance	Jutkina et al., 2016
3	2016	High levels of antibiotic resistance genes and their correlations with bacterial community and mobile genetic elements in pharmaceutical wastewater treatment bioreactors	Tao et al., 2016
4	2016	Reduction of antibiotic resistance genes in municipal wastewater effluent by advanced oxidation processes	Zhang et al., 2016
5	2017	Removal of antibiotic resistance genes from wastewater treatment plant effluent by coagulation	Li et al., 2017
6	2017	Metagenomic analysis reveals wastewater treatment plants as hotspots of antibiotic resistance genes and mobile genetic elements	Guo et al., 2017
7	2017	Subinhibitory concentrations of disinfectants promote the horizontal transfer of multidrug resistance genes within and across genera	Zhang et al., 2017
8	2017	H_2_O_2_ and/or TiO_2_ photocatalysis under UV irradiation for the removal of antibiotic resistant bacteria and their antibiotic resistance genes	Guo et al., 2017
9	2017	Β-lactams resistance gene quantification in an antibiotic resistant *Escherichia coli* water suspension treated by advanced oxidation with UV/H_2_O_2_	Ferro et al., 2017
10	2018	The occurrence, maintenance, and proliferation of antibiotic resistance genes (ARGs) in the environment: influencing factors, mechanisms, and elimination strategies	Zhang et al., 2018
11	2018	Removal of antibiotic resistance genes in two tertiary level municipal wastewater treatment plants	McConnell et al., 2018
12	2018	Antibiotics in wastewaters: a review with focus on Oman	Al-Riyami et al., 2018
13	2018	Reduction of antibiotic resistant bacteria during conventional and advanced wastewater treatment, and the disseminated loads released to the environment	Jäger et al., 2018
14	2018	Solar photo-Fenton disinfection of 11 antibiotic-resistant bacteria (ARB) and elimination of representative AR genes. Evidence that antibiotic resistance does not imply resistance to oxidative treatment	Giannakis et al., 2018
15	2018	Antibiotic-resistance genes in waste water	Karkman et al., 2018
16	2019	Wastewater treatment by advanced oxidation process and their worldwide research trends	Garrido-Cardenas et al., 2019
17	2019	High clonal diversity of multidrug-resistant and extended spectrum beta-lactamase-producing *Escherichia coli* in a wastewater treatment plant	Aristizábal-Hoyos et al., 2019
18	2019	Solar photo-Fenton oxidation followed by adsorption on activated carbon for the minimisation of antibiotic resistance determinants and toxicity present in urban wastewater	Michael et al., 2019
19	2019	A review on Fenton process for organic wastewater treatment based on optimization perspective	Zhang et al., 2019
20	2019	Copper nanoparticles and copper ions promote horizontal transfer of plasmid-mediated multi-antibiotic resistance genes across bacterial genera	Zhang et al., 2019
21	2019	Continuous ozonation of urban wastewater: removal of antibiotics, antibiotic-resistant *Escherichia coli* and antibiotic resistance genes and phytotoxicity	Iakovides et al., 2019
22	2019	Effect of solar photo-Fenton process in raceway pond reactors at neutral pH on antibiotic resistance determinants in secondary treated urban wastewater	Fiorentino et al., 2019
23	2019	Simultaneous removal of antibiotics and antibiotic resistance genes from pharmaceutical wastewater using the combinations of up-flow anaerobic sludge bed, anoxic-oxic tank, and advanced oxidation technologies	Hou et al., 2019
24	2019	State of the art of tertiary treatment technologies for controlling antibiotic resistance in wastewater treatment plants	Pei et al., 2019
25	2019	Antibiotic resistance genes identified in wastewater treatment plant systems—a review	Pazda et al., 2019
26	2020	Recent advances in ozone-based advanced oxidation processes for treatment of wastewater—a review	Rekhate et al., 2020
27	2020	Advancements in the Fenton process for wastewater treatment	Xu et al., 2020
28	2020	Antimicrobial resistance screening and profiles: a glimpse from the South African perspective	Genthe et al., 2020
29	2020	Inactivation of antibiotic-resistant bacteria and antibiotic resistance genes by electrochemical oxidation/electro-Fenton process	Chen et al., 2020
30	2020	A critical review on the occurrence of resistomes in the environment and their removal from wastewater using apposite treatment technologies: limitations, successes and future improvement	Anthony et al., 2020
31	2020	Efficient inactivation of antibiotic-resistant bacteria and antibiotic resistance genes by photo-Fenton process under visible LED light and neutral pH	Ahmed et al., 2020
32	2020	Impact of disinfection processes on bacterial community in urban wastewater: should we rethink microbial assessment methods?	Di Cesare et al., 2020
33	2021	The abundance of genes encoding ESBL, pAmpC and non-β-lactam resistance in multidrug-resistant Enterobacteriaceae recovered from wastewater effluents	Fadare et al., 2021
34	2021	Profiling of emerging contaminants and antibiotic resistance in sewage treatment plants: an Indian perspective	Saxena et al., 2021
35	2021	Perspectives of the solar photo-Fenton process against the spreading of pathogens, antibiotic-resistant bacteria and genes in the environment	Polo-López et al., 2021
36	2021	A review on non-thermal plasma treatment of water contaminated with antibiotics	Magureanu et al., 2021
37	2021	Monitoring antibiotic resistance genes in wastewater treatment: current strategies and future challenges	Nguyen et al., 2021
38	2021	Photo-assisted electrochemical advanced oxidation processes for the disinfection of aqueous solutions: a review	García-Espinoza et al., 2021
39	2021	A review on disinfection technologies for controlling the antibiotic resistance spread	Herraiz-Carboné et al., 2021
40	2021	Metagenomic analysis of MWWTP effluent treated via solar photo-Fenton at neutral pH: effects upon microbial community, priority pathogens, and antibiotic resistance genes	Vilela et al., 2021
41	2021	Combat of antimicrobial resistance in municipal wastewater treatment plant effluent via solar advanced oxidation processes: achievements and perspectives	Starling et al., 2021
42	2021	Distribution of extracellular and intracellular antibiotic resistance genes in sludge fractionated in terms of settleability	Li et al., 2021
43	2021	Solar photon-Fenton process eliminates free plasmid DNA harboring antimicrobial resistance genes from wastewater	Vilela et al., 2021
44	2021	Advanced oxidation processes for water disinfection: features, mechanisms and prospects	Chen et al., 2021
45	2022	Limitations and future directions of application of the Fenton-like process in micropollutants degradation in water and wastewater treatment: a critical review	Ziembowicz et al., 2022
46	2022	Fate of antibiotic resistance genes (ARGs) in wastewater treatment plant: preliminary study on identification before and after ultrasonication	Rumky et al., 2022
47	2022	Roles of reactive oxygen species in antibiotic resistant bacteria inactivation and micropollutant degradation in Fenton and photo-Fenton processes	Ahmed et al., 2022
48	2022	Degradation of bacterial antibiotic resistance genes during exposure to non-thermal atmospheric pressure plasma	Courti et al., 2022
49	2022	Risks of antibiotic resistance genes and antimicrobial resistance under chlorination disinfection with public health concerns	Ma et al., 2022
50	2022	Simultaneous removal of antibiotic resistant bacteria and antibiotic resistance genes by molybdenum carbide assisted electrochemical disinfection	Fang et al., 2022
51	2022	Benchmarking tertiary water treatments for the removal of micropollutants and pathogens based on operational and sustainability criteria	De Boer et al., 2022
52	2022	Critical review of technologies for the on-site treatment of hospital wastewater: from conventional to combined advanced processes	Pariente et al., 2022
53	2023	Multivalent metal catalysts in Fenton/Fenton-like oxidation system: a critical review	Liu et al., 2023
54	2023	Origin of antibiotics and antibiotic resistance, and their impacts on drug development: a narrative review	Muteeb et al., 2023
55	2023	The fate and occurrence of antibiotic-resistant bacteria and antibiotic resistance genes during advanced wastewater treatment and disinfection: a review	Kalli et al., 2023
56	2023	Chlorine disinfection modifies the microbiome, resistome and mobilome of hospital wastewater—a nanopore long-read metagenomic approach	Rolbiecki et al., 2023
57	2023	Effective removal of antibiotic resistance genes by high-frequency electromagnetic field combined with chlorine disinfection	Shi et al., 2023
58	2023	The dynamics and removal efficiency of antibiotic resistance genes by UV-LED treatment: an integrated research on single- or dual-wavelength irradiation	Zhao et al., 2023
59	2023	Electrochemical disinfection may increase the spread of antibiotic resistance genes by promoting conjugal plasmid transfer	Li et al., 2023
60	2023	Synergistic removal of pharmaceuticals and antibiotic resistance from ultrafiltered WWTP effluent: free-floating ARGs exceptionally susceptible to degradation	Gajdoš et al., 2023
61	2024	Prevention and potential remedies for antibiotic resistance: current research and future prospects	Khan et al., 2024
62	2024	Recent advancement of eliminating antibiotic resistance bacteria and antibiotic resistance genes in livestock waste: a review	Pham et al., 2024
63	2024	Urban wastewater disinfection by iron chelates mediated solar photo-Fenton: effects on seven pathogens and antibiotic resistance transfer potential	La Mann et al., 2024
64	2024	Homogeneous vs. heterogeneous photo-Fenton elimination of antibiotic-resistant bacteria bearing intracellular or extracellular resistance: do resistance mechanisms interfere with disinfection pathways?	Decker et al., 2024
65	2024	Antibiotic resistance and aquatic systems: importance in public health	Lajqi Berisha et al., 2024
66	2025	Antibiotics in wastewater: exploring the sources, links to antibiotic resistance, and strategies for their removal	Rajak et al., 2025
67	2025	Antibiotics and antibiotic resistance genes in the environment: dissemination, ecological risks, and remediation approaches	Wu et al., 2025
68	2025	Fate of extracellular antibiotic resistant genes in wastewater treatment plants: characteristics, persistence, transformation, removal and potential risk	Li et al., 2025
69	2025	Chlorination-induced spread of antibiotic resistance genes in drinking water systems	Zhao et al., 2025
70	2025	Abundance changes and transfer mechanism of antibiotic resistance genes in water under sub-lethal disinfection	Zhang et al., 2025
71	2025	The fate of intracellular and extracellular antibiotic resistance genes during ultrafiltration-ultraviolet-chlorination in a full-scale wastewater treatment plant	Li et al., 2025

* Internal study ID refers to an internal identification code used for systematic review management only; it is not a reference citation.

**Table 3:** Most commonly detected antibiotics and their associated antibiotic resistance genes in wastewater treatment plants.

Antibiotic Class	Common Antibiotics	Resistance Genes (ARGs)	Sampling Site	Concentration Range (ng/L–μg/L)	Effluent Proportion (%)	Persistence/Risk Level	References
Aminoglycosides	Tobramycin, gentamicin, kanamycin	*aadA*, *aacA4*, *aadB*, *aadE*, *strB*	Sludge	10–500	5–10%	Moderate (high affinity for sludge)	[1,31]
Beta-lactams	Amoxicillin, ampicillin, penicillin	*blaCTX-M*, *blaTEM*, *blaOXA-A*, *blaSHV*, *mecA*	Influent, tertiary effluent, activated sludge	50–2500	15–25%	Low (high hydrolysis rate)	[1,33]
Macrolides	Clarithromycin, erythromycin, azithromycin	*ereA*, *ermB*, *ermC*, *erm43*, *mefC*, *mphG*	Influent and secondary effluent	50–1500	10–20%	Moderate	[10,34]
Fluoroquinolones	Ofloxacin, ciprofloxacin, norfloxacin	*qnrS*, *qnrC*, *qnrD*	Influent, secondary effluent, digested sludge	20–1200	10–20%	High (strong adsorption)	[1,31]
Sulfonamides	Sulfamethoxazole	*sul1*, *sul2*	Influent, secondary effluent, activated sludge	100–5000	30–45%	High (highly mobile)	[1,10]
Tetracyclines	Tetracycline	*tetA*, *tetB*, *tetE*, *tetG*, *tetH*, *tetS*, *tetT*, *tetX*	Influent, secondary effluent, anaerobic sludge	10–800	5–15%	High (bioaccumulative)	[31,34]

**Table 4:** Methodological characteristics and quality assessment of studies investigating ARGs in wastewater (WW) matrices.

Matrix Type	Replication (*n* ≥ 3)	PCR Inhibitor Control	Detection Method	Quality Score	Key Limitation	Reference
Real WW	Yes	Yes	qPCR/Metagenomics	High	Broad scope; specific removal mechanisms for some ARGs require more detail.	[1]
Real WW	Yes	Yes	qPCR (absolute quantification)	High	Focus on plasmid DNA; genomic DNA removal may vary.	[33]
Urban WW	Yes	Yes	qPCR	High	Combined process complexity makes scaling-up analysis difficult.	[34]
Real WW	Yes	Yes	High-throughput-qPCR/Metagenomics	High	High variability between different WWTP configurations.	[31]
Real WW	Yes	No	qPCR/Metagenomics	Medium	Meta-analysis nature; raw data rigor depends on primary sources.	[10]

**Table 5:** Comparative summary of photo-Fenton studies for antibiotic resistance control. Log reduction values are reported as originally stated in each study; where only percentage removal was reported, it is indicated as such.

Matrix	pH	Fe Dose	H_2_O_2_ Dose	Time (min)	Light Source	Target	Log Reduction	Key Limitations	Reference
Real urban wastewater	Neutral	20 mg/L	50 mg/L	30–100	Natural solar	ARB: *E. coli*, *Enterococcus*; iARG: *sul*1, *qnr*, blaTEM	ARB: total (LOD); iARG: poor (e.g., *sul*1 < 1 log)	Low efficacy on ARGs; iron precipitation at neutral pH	[68]
PBS/synthetic wastewater	Neutral (6.8–7.4)	2.8 mg/L	340 mg/L	10–30	LED (visible)	ARB: *E. coli* DH5α; eARG: *tet*A, *bla*TEM-1; iARG: *tetA*, *bla*TEM-1	ARB: 6.17 log; eARG: 6.75–8.56 log; iARG: 0.52–1.02 log	Very high H_2_O_2_ dose required for iARGs; lab scale only	[70]
Ultrapure/synthetic wastewater	6.1–6.5	1 mg/L	10–20 mg/L	90–120	Simulated solar	ARB: 11 strains (e.g., *S. aureus*); iARG: *bla*CTX-M-9	ARB: total inactivation; iARG: rapid destruction	Risk of ARG persistence in lysate after bacterial inactivation	[71]
Real/synthetic wastewater	Neutral	30 mg/L (intermittent)	50 mg/L	60	Solar simulator	eARG: plasmids (amp/kan resistance)	80% (real); 100% (synthetic)	Efficacy decreases significantly in real matrices due to interferents	[33]
Secondary urban wastewater	Neutral (Fe:IDS)	5.5 mg/L	50 mg/L	180	Solar	ARB: *E. coli*, *S. aureus*; i/eARG: *qnr*S, *tet*A, *intl*1	ARB: 1.4–4.3 log; ARG: reduced expression (0.001-fold), but *intl*1 slightly increased	Matrix alkalinity and chelant stability limit efficacy	[67]
Municipal WWTP effluent	Neutral (intermittent addition)	30 mg/L	50 mg/L	240	Solar simulator	ARB: WHO priority pathogens *; iARG: *sul*, *tet*, β-lactams	ARB: total removal (metagenomics); ARG: 60% (*sul*, *tet*) to 100% (β-lactams)	Selective enrichment of Proteobacteria after treatment	[33]
Synthetic/real wastewater	Neutral (intermittent addition)	30 mg/L	50 mg/L	60	Solar simulator	eARG: free plasmids (pSB1A2, pSB1K3)	eARG: 100% (synthetic); ~80% (real)	Turbidity and carbonates in real matrix cause light scattering and radical quenching	[55]
Ultrapure water	Near-neutral	1 mg/L	10 mg/L	50–100	Solar simulator	ARB: *S. aureus* strains; iARG: *bla*CTX-M-9	ARB: 4 log; iARG: complete removal at 90 min	Resistance type (intra vs. extracellular) and cell wall thickness alter kinetics	[44]
CAS and MBR effluents	Acidic (2.8–2.9)	5 mg/L	50–100 mg/L	115	Solar	ARB: coliforms, heterotrophs; iARG: *sul*1, *qnr*S, *bla*TEM	ARB: total inactivation; iARG: below LOQ except *blaTEM* (persistent)	*bla*TEM relative abundance increased after 120 min of treatment	[34]

ARB: antibiotic-resistant bacteria, iARG: intracellular antibiotic resistance genes, eARG: extracellular antibiotic resistance genes, LOD: limit of detection, LOQ: limit of quantification, IDS: iminodisuccinate, CAS: conventional activated sludge, MBR: membrane bioreactor, PBS: phosphate-buffered saline, WWTP: wastewater treatment plant, WHO: world health organization, * https://www.who.int/publications/b/64088, accessed on 1 May 2026.

**Table 6:** Advantages and disadvantages of methods for antibiotic resistance genes (ARG) removal.

Methods	Advantages	Disadvantages	References
Chlorination	Low cost, inactivates enzymes, and causes cell death	Exerts selective pressure on antibiotic resistance bacteria (ARB) and promotes the spread of intracellular and extracellular ARGs	[5,33,46]
Ultraviolet (UV) radiation	Damages the DNA of microorganisms, causing death	Exposure to low doses increases the abundance of ARGs; high energy consumption	[29,41,48]
Ozone	Strong oxidant, attacks ARGs non-selectively	Expensive; leads to the formation of toxic byproducts; does not prevent the regrowth of bacteria and resistant genes	[51]
Electrochemical oxidation	Simple operation, lower energy consumption, and reduced environmental impact	Sublethal conditions may promote HGT (horizontal gene transfer)	[25,60]
Photocatalysis	Good results at laboratory scale	Possible inactivation and release of the TiO_2_ catalyst into the environment; higher costs	[56]
Fenton	Easy operation, high degradation of toxic compounds, easy access to reagents, and low cost	Limited by acidic operational conditions (pH 3)	[34,65]
Photo-Fenton	Increases •OH generation; efficient in removing intracellular and extracellular ARGs and ARB	Requires acidic pH; chelating agents are used, which can be toxic in some cases	[24,55]

**Table 7:** Removal efficiency, operational costs, and economic drivers for the treatment of antibiotic resistance genes (ARGs) in municipal effluents.

Treatment Method	ARGs Removal Efficiency	Estimated Operational Cost (USD/m^3^)	Economic Drivers/Considerations	References
Chlorination	Low to moderate	<0.10	Low cost; highly accessible infrastructure; risk of high chemical consumption for ARG degradation	[14,46]
Ozone (O_3_)	High	0.30–0.60	High energy consumption for ozone generation; efficient but requires high capital cost	[15,19]
Ultraviolet (UV) radiation	Moderate to high	0.15–0.40	Dependent on electricity prices and lamp maintenance; limited by water turbidity	[16,20]
Photo-Fenton (solar)	Very high	0.20–0.45	Competitive cost due to the use of solar energy; costs derive primarily from H_2_O_2_ and iron recovery.	[20,39]

## Data Availability

No new data were created or analyzed in this study. Data sharing is not applicable to this article.

## Abbreviations

The following abbreviations are used in this manuscript:

ARB	Antibiotic-resistant bacteria
AOPs	Advanced oxidation processes
ARGs	Antibiotic resistance genes
AMR	Antimicrobial resistance
CAS	Conventional activated sludge
DNA	Deoxyribonucleic acid
EDDS	Ethylenediamine disuccinic acid
EDTA	Ethylenediaminetetraacetic acid
eARGs	Extracellular antibiotic resistance genes
HGT	Horizontal gene transfer
•OH	Hydroxyl radical
iARGs	Intracellular antibiotic resistance genes
IDS	Iminodisuccinate
LED	Light-emitting diode
LOD	Limit of detection
LOQ	Limit of quantification
MBR	Membrane bioreactor
NTA	Nitrilotriacetic acid
PBS	Phosphate-buffered saline
qPCR	Quantitative polymerase chain reaction
UV	Ultraviolet
WWTPs	Wastewater treatment plants
WHO	World Health Organization
